# Anti-Jo1 Syndrome: Understanding a Rare Cause of Interstitial Lung Disease

**DOI:** 10.31138/mjr.33.4.437

**Published:** 2022-12-31

**Authors:** Arun Hegde, Vikas Marwah, Shrinath V, Robin Choudhary, Virendra Malik

**Affiliations:** 1 Department of Respiratory Medicine, AICTS, Pune, India,; 2 Department of Rheumatology, Command Hospital, Lucknow, India,; 3 Department of Radiology, AICTS, Pune, India

**Keywords:** anti-synthetase syndrome, idiopathic inflammatory myositis, anti-Jo1 antibody, interstitial lung disease

## Abstract

**Background:**

Anti-Jo1 syndrome is one of the most common amongst the various anti synthetase syndromes (ASS), which forms a subgroup of the idiopathic inflammatory myositis (IIM). It is characterised by myositis, interstitial lung disease (ILD), fever, Raynaud’s phenomenon, and mechanic’s hands; associated with the presence of anti-Jo1 antibodies in serum. Being an orphan disease, the clinical diagnosis is often delayed.

**Materials and methods:**

In this retrospective study, all patients diagnosed as Anti-Jo1 syndrome, from two tertiary care hospitals in Western Maharashtra, between 01 January 2019 – 31 December 2020, were enrolled. The parameters studied included demographic data, clinical features at presentation, laboratory parameters, spirometry, and radiographic findings, along with treatment instituted.

**Result:**

A total of 17 patients (8 males, 9 females) qualified for inclusion in the study. The mean age of diagnosis was 40 (±13) years with mean time to diagnosis being 2 years (± 0.6 years), from first clinical presentation. The most common presenting symptoms encountered were arthritis (n = 12, 70.5%), fever (n = 16, 70.5%), myositis (n=11, 64.7%) and breathlessness (n=10, 58.8%).10 patients had ILD at presentation on high resolution computerised tomography of chest (n=10, 58.8%) with restrictive lung defect on spirometry. Six patients required induction of immunosuppression using pulse methylprednisolone (n=6) and Rituximab (n=6), while 11 were managed with oral steroids. Mycophenolate mofetil (n=10) and Azathioprine (n=7) were used as maintenance immunosuppression.

**Conclusion:**

Anti-Jo1 syndrome is a myositis syndrome, presenting with a multitude of clinical features. Steroids and disease modifying anti rheumatic drugs form mainstay of therapy.

## INTRODUCTION

Anti-synthetase syndrome (ASS) is a clinical subset of idiopathic inflammatory myositis (IIM), manifesting with various combinations of fever, myositis, arthritis, interstitial lung disease (ILD), Raynaud’s phenomenon and mechanic hands, along with the presence of anti-amino-acyl-tRNA synthase (anti-ARS) antibodies in the serum. Anti Jo 1 syndrome is the commonest amongst the subset of ASS, with anti-Jo 1 antibody being the most common anti-ARS antibody. The disease commonly manifests in the fifth decade of life.^1–3^ Certain Indian studies have observed the commonest clinical manifestations to be arthritis (59%), myositis (59%), fever (41%), proximal muscle weakness (41%) and ILD (52%); out of which ILD contributes significantly to mortality and morbidity.^4^

The American, European Network of Antisynthetase Syndrome (AENEAS) collaborative group observed a mean diagnostic delay of ten years amongst patients who did not present with the complete triad of arthritis, myositis and ILD.^5^ Considering the heterogeneous nature of the disease, we attempted to study the demographic profile, clinical features, spirometry findings, radiological ILD patterns and management strategies, amongst patients with Anti-Jo1 syndrome presenting to two different tertiary care hospitals in Western India

## METHODS

### Study design

This was a retrospective study.

### Study setting

The study was carried out at two tertiary care hospitals located in Western Maharashtra.

### Study duration

01 Jan 2019 to 31 Dec 2020.

### Study population

The study population comprised of all patients who presented to the outpatient departments with a combination of complaints comprising arthritis, breathlessness on exertion, Raynaud’s phenomenon, mechanic’s hands, and fever (not attributable to other causes).

### Inclusion criteria

Patients who were diagnosed with ASS, as per Connor’s criteria were included in the study.^6^ (**Table 1**)

**Table 1.** Connor et al. 2010 criteria for diagnosis of anti-synthetase syndrome.^8^

**Required**	**Presence of an anti-aminoacyl tRNA synthetase antibody**
One or more of the following clinical features	Raynaud’s phenomenon Arthritis Interstitial Lung Disease Fever (not attributable to other causes) Mechanic’s hands

### Exclusion criteria

Patients with arthritis and ILD, but not positive for anti-Jo1 antibody.

### Study variables

Demographic data, clinical features on presentation, laboratory data, spirometry finding, radiographic findings, and treatment instituted were taken from rheumatology and respiratory medicine OPD records. Patients with clinical suspicion underwent evaluation for anti-nuclear antibody (ANA) by indirect immunofluorescence assay (IIF), extractable nuclear antigens (ENA) using enzyme linked immunosorbent assay (ELISA), and rheumatoid factor (RF), using nephelometry. Patients also underwent testing for muscle enzymes including creatine kinase (normal range: 24 - 173 U/L), lactic dehydrogenase (normal range: 20 - 350 U/L), aminotransferase alanine (normal range: 10–36 U/L), and aminotransferase aspartate (normal range: 10–36 U/L). Acute phase reactants like C-reactive protein (normal range: < 7mg/dl), erythrocyte sedimentation rate (normal range: 0 – 30 mm/hr), complement levels (C3 normal range: 90–180 mg/dl, C4 normal range: 15–45 mg/dl) were carried out for nine out of the total seventeen patients.

Patients satisfying Connor’s criteria for anti-synthetase syndrome were screened for ILD with high resolution computed tomography (HRCT) and spirometry. HRCT on full inspiration was taken from lung apices to base with patient in supine position. Axial, coronal, and sagittal sections of 1mm thickness were obtained. ILD pattern on HRCT were classified as per joint American Thoracic Society and European Respiratory Society classification of idiopathic interstitial pneumonia.^7,8^ Patients were considered to have ILD if there were features of inter/intra lobular septal thickening, ground glass opacities, honey combing or traction bronchiectasis with or without clinical symptoms.^3^ Spirometry was conducted as per 2019 ATS and ERS technical statement.^9^ Impairment of pulmonary function was categorised as mild, moderate, and severe when FEV_1_ is ≥ 70%, 50 – 69% and < 50% respectively.^10^

### Statistical methods

Categorical variables in the data have been reported as numbers and percentages, and continuous variables as mean (standard deviation) or median (range).

### Ethical clearance

The institutional ethics committee approved the study. Waiver for consent was taken from ethics committee.

## RESULTS

A total of 17 patients were diagnosed with ASS during the time period of the study and were included. Eight were male (47%) and nine were female (53%). Mean age at diagnosis was 40 (±13) years with mean time to diagnosis being 2 (± 0.6) years, from first clinical presentation. Analysis of patient’s symptoms on presentation (**Table 2**) showed that 12 (70.5%) patients had fever on presentation, 14 (82.4%) patients had musculoskeletal symptoms (out of which 12 had arthritis and 11 had myositis), 11 (64.7%) had cutaneous signs while 10 (58.8%) cases had respiratory symptoms. The most common presenting symptoms were arthritis (n = 12, 70.5%), fever (n = 16, 70.5%) myositis (n=11, 64.7%) and breathlessness (n=10, 58.8%). Other findings included Raynaud’s phenomenon (n= 10, 58.8%), mechanic’s hand (n= 5, 29.4%), heliotrope rash (n= 4, 23.5%), Shawl sign (n= 1, 5%) and malar rash (n = 1, 5%). The classic triad of arthritis, myositis and ILD was seen only in 4 (23.5%) patients. The most common duad of symptoms was arthritis and myositis (n= 9, 53%).

**Table 2.** Clinical characteristics of patients with anti-synthetase syndrome (n = 17).

	**n (%) except where specified**

Age, mean ± SD in years	40 ± 13

Male to female ratio	8:9

Clinical features	
1. Constitutional	12 (70.5)
- Fever	12 (70.5)
2. Musculoskeletal	14 (82.4)
- Arthritis	12 (70.5)
- Myositis	11 (64.7)
3. Pulmonary	10 (58.8)
- Shortness of breath	10 (58.8)
4. Raynaud’s phenomenon	10 (58.8)
5. Cutaneous	11 (64.7)
- Mechanic’s hands	5 (29.4)
- Heliotrope rash	4 (23.5)
- Shawl sign	1 (5)
- Malar rash	1 (5)
- Gottron papule	4 (23.5)
6. Serositis	1 (5)

All patients underwent an anti-nuclear antibody (ANA) test, using immunofluorescence (IIF), out of 17 patients 15 (88%) had a positive ANA. The most common staining pattern was cytoplasmic (n= 10, 67%), followed by speckled (n = 5, 33%). All patients were positive for anti – Jo1 antibodies. Apart from anti-Jo1, patients also tested positive for anti-Ro 52 (n = 8, 47%), Ro 60 (n = 5, 29%) and AMA M2 (n = 1, 5%)

Baseline spirometry was carried out in all patients, out of which ten showed restrictive defect and seven had normal spirometry. All 10 patients who presented with shortness of breath had restrictive defect on spirometry. Six patients had mild restriction, moderate and severe restriction were noted in two patients each. HRCT was done for all the patients. Out of the 17 patients, 10 patients who had shortness of breath were diagnosed with ILD on HRCT. The most common pattern was NSIP (n = 6, 60%) followed by UIP (n = 4, 40%). All the ten patients who had shortness of breath were diagnosed with ILD on HRCT. The seven patients who didn’t have shortness of breath had a normal HRCT finding.

All patients were managed with induction phase of immunosuppression followed by maintenance immunosuppression. Induction was achieved using pulse methylprednisolone and rituximab (both, n=6) or oral steroids alone (n=11). The dose used for glucocorticoid pulse therapy was IV methylprednisolone 30 mg/kg/day for three days. Rituximab was given as two 375 mg/m^2^, IV doses, spaced over two weeks and oral steroid induction was with tablet prednisolone 1mg/kg daily. Tapering dose of oral prednisolone along with disease modifying antirheumatic drugs (DMARDs) like mycophenolate mofetil (MMF) (n=13) or azathioprine (n=4) were used for maintenance therapy.

## DISCUSSION

Our study comprised of patients with anti-Jo1 syndrome, a subset of ASS. Due to the high frequency of non-specific signs associated with anti-Jo1 syndrome, criteria proposed by Connor et al, ae used the world over, for diagnosis.^6^ The same criteria were applied to patients in our study. The mean age at presentation in our study (40 ± 13 years) was comparable to the findings of a study by Kumar et al. (40 ± 9.2).^4^ The largest international study on patients with anti-Jo1syndrome, revealed mean age at presentation to be 53 years.^11^ The Indian population seems to have an earlier onset of symptoms when compared to American and European population based on these results. The exact reason for the same is not known. The disease has a predisposition for the female sex, as was observed by Kumar et al, and in the AENEAS cohort.^4,11^ Our study showed an equal sex predisposition, which could be due to a small sample size.

The most common presenting symptoms in our study were fever and arthritis, which was similar to the findings of Kumar et al. The classic triad of ILD, myositis and arthritis were less frequent in Indian patients on initial presentation, as per our study (23.5%) and that by Kumar et al, (15%). Our study observed that 53% presented with two manifestations amongst the classic triad of symptoms (most common being arthritis along with myositis), while 17.6% had just one feature (most common being arthritis) and 1 patient was diagnosed with ASS without exhibiting even one amongst the classic triad of clinical features. This aforementioned patient was a 29-year-old male who presented with Raynaud’s phenomenon without any features of arthritis, myositis and ILD and who on evaluation was found to be anti-Jo-1 positive by immunoblot assay. The dermatological features which were observed in these patients were Mechanic’s hands, heliotrope rash, shawl sign, Gottron papules, and malar rash. Dermatological manifestations on presentation were seen in 11 (64.7%) cases in our study as compared to 37% cases in Kumar et al. study. The most common dermatological manifestation noted were mechanic’s hand and Gottron papules as per our study and the study by Kumar et al. This shows the heterogenicity of clinical presentation of anti-Jo 1 syndrome. Patients who have ASS antibodies can present with combinations of the classic triad, or may even present with a single non-specific minor clinical feature. Even though musculoskeletal complaints and fever comprise the chief presenting complaints in Indian patients; as per our study (82.4%, 70.5%) and as per the study by Kumar and associates, (59% and 41%), pulmonary manifestations are the most important determinants of morbidity.^4^ The current study demonstrated a prevalence of ILD of 58.8% in anti-Jo1 positive patients at presentation, as compared to 52% in Kumar et al and 50% in AENEAS cohort. These studies reveal that about 50% of patients who are positive for anti-Jo 1 antibodies have ILD at presentation. The typical findings on HRCT chest are bibasilar fibrosis, ground glass opacities, interlobular reticulations, and traction bronchiectasis. The most common radiological pattern observed was NSIP (our study [60%], Kumar et al. [81%]), followed by UIP (our study [40%] and Kumar et al. study [9%]). There was no organising pneumonia pattern on HRCT in our study but Kumar et al observed 9% of anti-Jo 1 patients to be having an organising pneumonia pattern. The most common functional pattern was a restriction defect on spirometry with all patients diagnosed to have ILD showing a restrictive pattern and none showing obstructive defect. Spirometry, 6-minute walk test (6MWT) and HRCT were used to screen asymptomatic patients as interstitial involvement could often be subclinical. In our study the 7 patients who didn’t have breathlessness on exertion had normal HRCT, 6-MWT and spirometry. Comparison of our clinical finding with existing Indian and international data is given in **Table 4**.

**Table 3.** Laboratory characteristics of patients with anti-synthetase syndrome (n = 17).

**Test**	**n (%)**

Anti-Jo-1	17 (100)

ANA	15(88)
Pattern on IFA	
- Cytoplasmic	10 (67)
- Speckled	5 (33)

Muscle enzyme levels	
- Creatine kinase (U/L)	1698.5 ± 3486
- Lactic dehydrogenase (U/L)	682.3 ± 580.7
- Aminotransferase alanine (U/L)	80 ± 113.5
- Aminotransferase aspartate (U/L)	97.7 ± 141.5

HRCT	
- UIP	4 (40%)
- NSIP	6 (60%)

Spirometry	
- Restriction	10 (mild 6, moderate 2, severe 2)
- Normal	7

**Figure 1.**
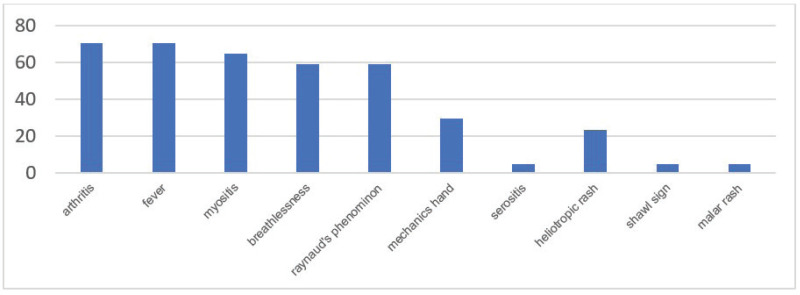
A bar chart showing the percentage of patients having the characteristic clinical symptoms and signs on presentation.

**Table 4.** Comparison of clinical presentation of anti-synthetase syndrome (anti-Jo1 positive) in the present study, a leading Indian study, and an international study.

	**Present study**	**Kumar et al. descriptive study (2019)**	**AENEAS study**
Total patients in study (anti-Jo1 positive)	17	27	225
Age of presentation	40 ± 13	40 ± 9.2	53 (42–63)
Male female ratio	1:1.1	1:4	1:3
Most common presenting symptom	Fever (70.5%) and arthritis (70.5%)	Arthritis (59%) and myositis (59%)	Arthritis (64%) and myositis (58%)
Patients who presented with classic triad	23.5%	15%	19.5%
ILD on presentation	58.8%	52%	50%
Arthritis on presentation	70.5%	59%	64%
Myositis on presentation	64.7%	59%	55.5%
Fever on presentation	70.5%	41%	-
Raynaud’s phenomenon on presentation	58.8%	19%	23%
Mechanic’s hands on presentation	29.4%	19%	18%

In our study, patients manifesting with severe ILD (hypoxic on presentation with extensive lung involvement) and those with elevated muscle enzyme levels were managed with steroid pulse therapy and rituximab, while the rest were managed with only oral steroids and MMF/azathioprine. There is very little literature on either the choice of drugs, or the duration of treatment for ASS. Corticosteroids have long been the corner stone for managing patients with IIM, and associated ILDs. When used alone it is rarely sufficient to control the disease activity with high incidence of ILD recurrence being reported with steroid monotherapy.^12^ A multicentric cohort study from India showed the prevalence of anti-Jo1 antibodies to be 10% among patients with idiopathic inflammatory myositis.^13^ Additional immunosuppressive agents are usually added in case of ILD, refractory myositis or as steroid sparing drugs. Although there is no consensus on the particular kind of immunosuppression use, DMARDs in form of azathioprine, MMF, tacrolimus, rituximab and cyclophosphamide are being commonly used for management of refractory myositis and ILD. Few retrospective case series have shown modest improvement in lung function with azathioprine.^14–16^ Significant improvement in lung function was demonstrated in a retrospective study of 125 patients with use of MMF in connective tissue disease associated ILDs.^17^ There are a significant number of retrospective studies which have demonstrated the benefit of rituximab in managing ILD associated with ASS.^18–28^ These studies have demonstrated a significant improvement of lung function test and radiological clearance in patients managed with rituximab. Our management protocol was similar to the one used in the studies by Huang and Aggarwal.^12^

Our study had a few limitations. The patients were not screened for pulmonary artery hypertension. MRI thighs and electromyography couldn’t be carried out in all patients. Patients were not screened for other myositis specific antibodies apart from anti-Jo-1.

**Figure 2.**
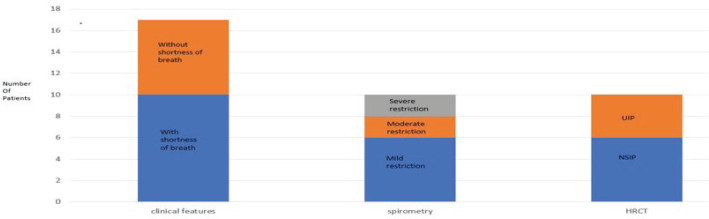
A compound-bar chart showing the clinical, spirometry and HRCT finding of ILD in patients with anti-synthetase syndrome.

## CONCLUSION

Anti-Jo1 syndromes are a subset of IIM that can have a heterogenous presentation. ILD is one of the major presenting symptoms and the most common cause of morbidity and mortality in the disease. As the disease entity is rarely diagnosed, a high index of suspicion needs to be entertained while assessing patients with a combination of symptoms comprising of arthritis, myositis, Raynaud’s phenomenon, and mechanic’s hand with underlying ILD.
